# Awareness of Orthodontic Treatments among School Teachers of Two Cities in Iran

**DOI:** 10.5681/joddd.2012.006

**Published:** 2012-03-13

**Authors:** Ali Rafighi, Saeid Foroughi Moghaddam, Mahasti Alizadeh, Hadi Sharifzadeh

**Affiliations:** ^1^Assistant Professor, Department of Orthodontics, Faculty of Dentistry, Tabriz University of Medical Sciences, Tabriz, Iran; ^2^Postgraduate Student, Department of Orthodontics, Faculty of Dentistry, Tabriz University of Medical Sciences, Tabriz, Iran; ^3^Associate Professor, Department of Community Medicine, Faculty of Medicine, Tabriz University of Medical Sciences, Tabriz, Iran; ^4^Dentist, Private Practice, Saqqez, Iran

**Keywords:** Awareness, orthodontic treatment, teachers

## Abstract

**Background and aims:**

Since most orthodontic patients are children and adolescents, it is believed that teachers can help find these patients and make them aware of their orthodontic problems. Therefore, teachers are expected to have proper knowledge about these problems. The aim of this study was to determine teachers’ awareness about orthodontic treatments in Tabriz and Saqqez and compare it in different educational levels.

**Materials and methods:**

A questionnaire was given to 384 teachers (204 in Tabriz and 180 in Saqqez) in randomly selected schools of different levels. The questionnaire had 17 questions in 3 sections, including general information, age, and costs of orthodontic treatments. The teachers’ awareness in two cities was analyzed with independent t-test and in edu-cational levels with one-way ANOVA.

**Results:**

About 94% of the participants believed orthodontic treatment consisted of arrangement of teeth with special braces. However, there was no significant difference between the two cities in general information (p=0.23) and the age suitable for orthodontic treatment (p=0.53). The differences in the teachers’ awareness in three educational levels were not statistically significant between the two cities (p=0.23).

**Conclusion:**

Awareness of teachers about orthodontic treatment in Saqqez and Tabriz was not sufficient and different; 75% of the participants believed that high cost of orthodontic treatment is the main problem.

## Introduction

Tooth malalignment may cause a number of problems, including social discrimination due to different facial appearance, oral function problems such as difficulties in jaw movements (lack of muscle coordination or pain), temporomandibular joint disorder (TMD), problems of masticatory system, swallowing or speech, and increased susceptibility to trauma, periodontal disease or caries. ^1^

However, almost always, orthodontic treatment is an elective one but it can be useful in cases of psycho-social, developmental, functional, and oral health problems.^2^

Facial appearance is one of the most important physical characteristics in the development of one's self-confidence; therefore, it seems that people who are satisfied with their own appearance, have more self-confidence in comparison with others.^3^ The most important motivation for orthodontic treatment is improvement in dentofacial esthetics.^4 , 5^

An increase in the number of orthodontic treatments has been seen in most industrialized countries in recent decades.^6 , 7^Demand for orthodontic treatment is influenced not only by the malocclusion prevalence and severity but also by sex, socioeconomic status, and ethnic origin, as well as availability and funding of orthodontic services.^6 - 8^ For instance, it has been observed that girls, in general, undergo orthodontic treatment more frequently than boys.^9^

The norms for satisfactory, ordinary, and attractive facial appearance are mostly determined by social forces. Social context and cultural environment have equal force as objective characteristics in identification of abnormal, unsatisfactory, and disfiguring dentofacial appearance by children and their parents. Although dissatisfaction with dentofacial appearance is influenced by the severity of malocclusion, subjects vary in recognition and perception of dental features.^10 , 11^

Children and adolescents comprise the bulk of orthodontic patients. Consequently, their guardians may have an important role in treatment commencement and compliance until the end of it. It has been shown that the most powerful single factor of motivation for orthodontic treatment is parents.^12 , 13^

School teachers are another group with a close relationship with children and adolescents, and students are widely influenced by them. Ehizele et al^14^ concluded that effective dental education of children can be accomplished by a multi-disciplinary approach in which teachers can play the role of oral health educators but only if they have good knowledge, attitude and practice of oral health; therefore, primary school teachers can serve as oral health educators after systematic training to increase their knowledge of oral health.^ P ^Similarly, school teachers are probably one of the important factors influencing demand for orthodontic treatment and subsequent compliance problems. The aim of this study was to evaluate and compare school teachers’ knowledge about orthodontic treatment in two cities (Tabriz and Saqqez) in Iran.

## Materials and Methods

Two cities—Tabriz and Saqqez—were selected as survey sites. The selection was based on apparent differences in cultural, societal, and economic status, population and availability of orthodontic services, such as dentistry faculty and orthodontists. Tabriz has a school of dentistry and many orthodontists in contrast to Saqqez. To receive orthodontic services the people of Saqqez should take trips to Tabriz or other distant cities.

A total of 384 subjects (204 in Tabriz, 180 in Saqqez) were selected and then divided equally into three groups based on educational levels (elementary, guidance, and high school); as a result, there were 68 subjects in each level in Tabriz and 60 in Saqqez. The schools were selected randomly from a list which included the names of all the schools of each city sorted by their regions.

A questionnaire including general information, proper age and costs of orthodontic treatment was prepared and the teachers were given 15 minutes to fill it, which was a short time to gather information from other sources. Therefore, the participants answered the questionnaire by their own knowledge.

The questionnaire consisted of 17 questions in 3 sections, including general information, proper age, and costs of orthodontic treatment.

Face and content validity of the questionnaire was confirmed by the specialists and according to the articles on social awareness of the orthodontic treatment. Each correct answer was given 1 point and 0 point was given for each wrong answer.

Independent t-test was used to compare the awareness between the two cities, and one-way ANOVA was used to make a comparison between educational levels in each city.

## Results

The questionnaire had good reliability according to the Cronbach's Alpha coefficient (0.62). In Tabriz, 177, 193 and 201 teachers out of 204 answered all the questions of general information, proper age and treatment costs, respectively; 145, 171, and 177 out of 180 did the same in Saqqez.

Descriptive statistical analysis (mean ± SD, frequency) was carried out using SPSS software for general information, age and cost of treatment. Kolmogrov-Smirnov test showed normal distribution of data.

Mean scores for general information were 55.3±17.0 in Tabriz and 53.0±15.7 in Saqqez, with no statistically significant differences (p=0.23). In both cities, mean scores were higher for general information among teachers of the guidance school but there was no statistically significant differences between educational levels in both cities (p=0.26).

Mean scores for knowledge about proper age for orthodontic treatment were 37.8±30.0 in Tabriz and 39.3±32.0 in Saqqez, with no statistically significant differences (p=0.53).

A total of 75% of teachers believed that orthodontic treatment is too expensive to be afforded easily; 17% were unaware about the costs and 5% believed that the costs are reasonable.

In general, there were no significant differences in awareness of orthodontic treatment between the two cities (p=0.23). The answers to some important parts of the questionnaire are summarized in Table 1.

**Table 1:** Percentages (%) of some answers to the questionnaire

Providers' answer	Tabriz	Saqqez	Mean
Have heard about orthodontics	85.0%	73.0%	79.0%
Orthodontics is not only for cosmetic purposes	68.0%	59%	65.3%
Teeth can be moved in the bone	66.0%	60.0%	64.4%
Orthodontic treatment cannot be rendered at any age	54.0%	45.0%	51.0%
Age is an important factor in beginning and duration of orthodontic treatment	75.0%	68.0%	72.0%
Orthodontic treatment should begin in young ages	61.0%	41.0%	52.8%
Orthodontic treatment can be rendered at any age	25.0%	40.0%	32.2%
Orthodontic treatment is expensive	80.4%	68.7%	75.0%
Had a relative under orthodontic treatment	47.0%	32.0%	41.0%

## Discussion

This study was designed to determine school teachers' awareness about orthodontic treatments in Tabriz and Saqqez and compare it in different educational levels. A questionnaire was given to 384 subjects in different educational levels (204 in Tabriz and 180 in Saqqez) in randomly selected schools. The questionnaire consisted of 17 questions in 3 sections, including general information, age, and costs of orthodontic treatment.

There were no statistically significant differences in general information and awareness about the proper age of orthodontic treatment between the two cities. A large number of subjects in both cities had heard about orthodontics. However, some claimed that they knew about the alignment of the teeth by the braces but not the scientific nomenclature of "orthodontics". Most participants’ correct answer to the meaning of "orthodontics" (94.7%) could be attributed to the questionnaire itself, because they became familiar with the word by filling the form which reminded them of their relatives' experiences of orthodontic treatment.

Although most of the subjects knew the meaning of "orthodontics", only half had adequate knowledge about orthodontic treatments, its durations, and proper age for orthodontic treatment, with no significant differences between the two cities. Most of the teachers believed that orthodontic treatment was expensive, which was its most important disadvantage.

Educational level which may lead to differences in experience, knowledge of the teachers and little difference in teachers' income (teachers of higher educational levels also have higher income) had no effect on the awareness about orthodontic treatment. Although the two cities had differences in availability and use of orthodontic services, the attitudes were almost the same about malocclusion and orthodontic treatment.

Most of the teachers believed that orthodontic treatment should be rendered in young ages (61% in Tabriz, 41% in Saqqez). Their deduction was that if the treatment is rendered at a young age, it will be more successful and also easier.

More teachers in Saqqez believed that tooth movement will be harmful, which might be attributed to low frequency of orthodontic treatment in the city and among their relatives. Awareness of the teachers in both cities about possibility of tooth movement and probability of harmful side effects was not adequate.

Oshagh et al^15^ showed that parents' awareness of children's orthodontic problems can be increased by means of information leaflets. Therefore, maybe lack of information about orthodontics in the media or any other type of it, which did not differ much between the two cities despite other differences, might be attributed to lack of knowledge about orthodontics among the school teachers. Importance of an orthodontic information package has been emphasized by Anderson et al.^16^

In addition, maybe if the teachers had knowledge about the installment of orthodontic treatment costs, or if the insurance companies had paid some of it, the cost would no longer be the greatest factor precluding treatment. Engagement of insurance companies could play the role of information media as well.

We recommend that studies be carried out on the influence of any type of information media on orthodontic treatment awareness to find the appropriate methods of informing people of the subject.

## Conclusion

Differences in cultural, societal, economic, population and availability of orthodontic services resulted in no significant differences in general information about orthodontics, proper age of treatment, and costs of treatment. Maybe lack of information by any type of media contributed to inadequate awareness about orthodontics in both cities.
